# Treatment for Opioid Use Disorder: Population Estimates — United States, 2022

**DOI:** 10.15585/mmwr.mm7325a1

**Published:** 2024-06-27

**Authors:** Deborah Dowell, Samantha Brown, Shiromani Gyawali, Jennifer Hoenig, Jean Ko, Christina Mikosz, Emily Ussery, Grant Baldwin, Christopher M. Jones, Yngvild Olsen, Naomi Tomoyasu, Beth Han, Wilson M. Compton, Nora D. Volkow

**Affiliations:** Corresponding author: Deborah Dowell, ddowell@cdc.gov.; ^1^Division of Overdose Prevention, National Center for Injury Prevention and Control, CDC; ^2^Substance Abuse and Mental Health Services Administration, Rockville, Maryland; ^3^National Institute on Drug Abuse, Bethesda, Maryland.

**Summary**

**What is already known about this topic?**

Although medications for opioid use disorder (OUD) substantially reduce mortality, they are underused.

**What is added by this report?**

In 2022, among the 4% of U.S. adults who needed OUD treatment, only 25% received recommended medications. A larger percentage (30%) received treatment without medications. Higher percentages of White than Black or African American or Hispanic or Latino adults received any treatment. Higher percentages of men than women and of adults aged 35–49 years than other adults received medications.

**What are the implications for public health practice?**

Expanded communication about the effectiveness of medications for OUD is needed. Clinicians and other treatment providers should offer or arrange evidence-based treatment, including medications for OUD. Pharmacists and payors can support making these medications available without delays.

## Abstract

In 2022, 81,806 opioid-involved overdose deaths were reported in the United States, more than in any previous year. Medications for opioid use disorder (OUD), particularly buprenorphine and methadone, substantially reduce overdose-related and overall mortality. However, only a small proportion of persons with OUD receive these medications. Data from the 2022 National Survey on Drug Use and Health were applied to a cascade of care framework to estimate and characterize U.S. adult populations who need OUD treatment, receive any OUD treatment, and receive medications for OUD. In 2022, 3.7% of U.S. adults aged ≥18 years needed OUD treatment. Among these, only 25.1% received medications for OUD. Most adults who needed OUD treatment either did not perceive that they needed it (42.7%) or received OUD treatment without medications for OUD (30.0%). Compared with non-Hispanic Black or African American and Hispanic or Latino adults, higher percentages of non-Hispanic White adults received any OUD treatment. Higher percentages of men and adults aged 35–49 years received medications for OUD than did women and younger or older adults. Expanded communication about the effectiveness of medications for OUD is needed. Increased efforts to engage persons with OUD in treatment that includes medications are essential. Clinicians and other treatment providers should offer or arrange evidence-based treatment, including medications, for patients with OUD. Pharmacists and payors can work to make these medications available without delays.

## Introduction

In 2022, more opioid-involved overdose deaths (81,806) were reported in the United States than in any previous year.* Medications for opioid use disorder (OUD) include buprenorphine, methadone, and extended-release naltrexone. These medications, especially buprenorphine and methadone, substantially reduce overdose-related and overall mortality but are markedly underused (*1*,*2*). Using an OUD cascade of care framework adapted from HIV care delivery improvement efforts (*2*), National Survey on Drug Use and Health (NSDUH) data were used to estimate and characterize U.S. adult populations who 1) need OUD treatment, 2) perceive a need for OUD treatment, 3) receive any OUD treatment, and 4) receive medications for OUD.

## Methods

### Data Source

NSDUH collects substance use and substance use disorder (SUD) treatment information through in-person and web-based interviews among a nationally representative sample of civilian, noninstitutionalized persons aged ≥12 years in the United States.^†^ Data from 56,610 adults aged ≥18 years (weighted interview response rate = 48.0%) participating in the 2022 NSDUH were analyzed to estimate numbers and percentages of adults who needed OUD treatment, perceived a need for OUD treatment, received any OUD treatment, and received medications for OUD in the past year.

### Definitions

The *Diagnostic and Statistical Manual of Mental Disorders, Fifth Edition* (DSM-5)^§^ describes OUD as “a problematic pattern of opioid use leading to clinically significant impairment or distress.” Needing OUD treatment was defined as meeting DSM-5 criteria for mild or moderate OUD (two to five symptoms) or severe OUD (six or more symptoms) or receiving OUD treatment during the preceding year. Persons receiving OUD treatment without meeting OUD criteria in the preceding year were assumed to have had an active OUD (i.e., to have had symptoms meeting criteria for OUD) in the past and to have successfully treated OUD with continued treatment. Respondents needing but not reporting OUD treatment in the previous year were asked whether they sought or thought they should receive OUD treatment; affirmative responses were coded as perceiving need for treatment. Receipt of OUD treatment was defined as receiving treatment for OUD or receiving treatment for an unspecified SUD along with reporting opioid use, with the assumption that the unspecified SUD was OUD. Receipt of medications for OUD was defined as taking medication in the past year prescribed to help reduce or stop opioid use (e.g., buprenorphine, methadone, or naltrexone).

### Statistical Analysis

Weighted prevalence estimates and 95% CIs were calculated overall and by sociodemographic-, health-, and substance-related characteristics. Log-linear chi-square tests of independence assessed overall differences between subgroups, followed by pairwise comparisons using *t*-tests. Analyses were conducted using SAS-callable SUDAAN (version 11.0; RTI International) to account for NSDUH’s complex design and sampling weights. This activity was reviewed by CDC, deemed not research, and conducted consistent with applicable federal law and CDC policy.^¶^

## Results

In 2022, an estimated 3.7% of U.S. adults (9,367,000) needed OUD treatment (Figure) (Table). Among these, 55.2% (5,167,000) received OUD treatment, and 25.1% (2,353,000) received medications for OUD (Figure). Most adults who needed OUD treatment either did not perceive that they needed it (42.7%) or received treatment that did not include medications for OUD (30.0%) (Figure).

**FIGURE:**
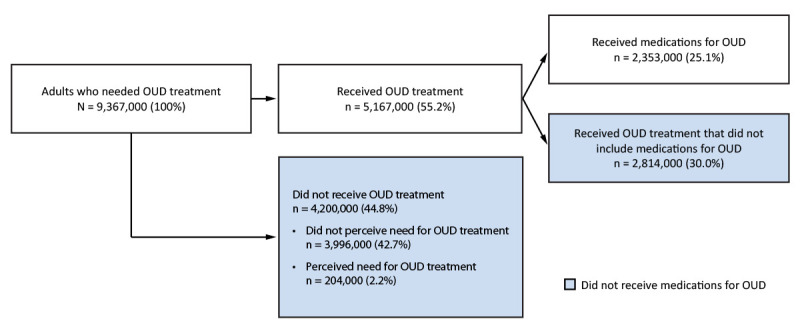
Estimated opioid use disorder treatment among adults aged ≥18 years — National Survey on Drug Use and Health, United States, 2022* **Abbreviation:** OUD = opioid use disorder. * Needing OUD treatment was defined as meeting *Diagnostic and Statistical Manual of Mental Disorders, Fifth Edition* criteria for OUD or receiving OUD treatment during the past year. Receiving OUD treatment was defined as receiving treatment for opioid use or receiving treatment for an unspecified substance along with reporting opioid use in the past year. Receiving medications for OUD was defined as having used medication in the past year prescribed to help reduce or stop opioid use. Examples of medications shown to respondents included methadone, buprenorphine or buprenorphine-naloxone, injectable buprenorphine, buprenorphine implants, naltrexone pills, and injectable naltrexone. Adults were classified as perceiving need for OUD treatment if they responded affirmatively to questions about whether they thought they should receive or sought drug use treatment in the past year. Numbers are weighted estimates rounded to the nearest thousand. Percentages are numbers (n or N) divided by the overall N (9,367,000) of adults who needed OUD treatment. Percentages are rounded to the nearest tenth and might not sum to 100% because of rounding.

**TABLE:** Estimated number* and percentage of adults aged ≥18 years who during the past year needed opioid use disorder treatment,^†^ received opioid use disorder treatment,^§^ or received medication for opioid use disorder^¶^ — National Survey on Drug Use and Health, United States, 2022

Characteristic	Needed OUD treatment^†^		Received OUD treatment among adults classified as needing OUD treatment^§^		Received medication for OUD among adults who received OUD treatment^¶^	
	Estimated weighted no.* (95% CI)	% (95% CI)	Estimated weighted no.* (95% CI)	% (95% CI)	Estimated weighted no.* (95% CI)	% (95% CI)
**Total**	**9,367 (8,603–10,196)**	**3.7 (3.4–4.0)**	**5,167 (4,756–5,571)**	**55.2 (50.8–59.5)**	**2,353 (2,077–2,634)**	**45.5 (40.2–51.0)**
**Sex**						
Female	4,623 (4,144–5,154)	3.5 (3.2–3.9)	2,461 (2,207–2,711)	53.2 (47.7–58.6)	971 (813–1,139)	39.5 (33.0–46.3)**
Male	4,744 (4,160–5,407)	3.8 (3.3–4.3)	2,706 (2,384–3,016)	57.0 (50.2–63.6)	1,381 (1,156–1,605)	51.0 (42.7–59.3)**
**Age group, yrs**						
18–25	770 (663–894)	2.2 (1.9–2.6)^††^	453 (391–512)	58.9 (50.8–66.5)	90 (62–128)	19.9 (13.7–28.1)^§§^
26–34	1,534 (1,292–1,818)	3.8 (3.2–4.5)	1,039 (887–1,171)	67.8 (57.8–76.3)	458 (365–555)	44.1 (35.1–53.4)^§§^
35–49	2,692 (2,359–3,069)	4.3 (3.8–4.9)	1,713 (1,538–1,875)	63.6 (57.1–69.7)	1,171 (1,055–1,275)	68.4 (61.6–74.5)^††^
≥50	4,371 (3,769–5,065)	3.7 (3.2–4.3)	1,962 (1,645–2,288)	44.9 (37.6–52.3)^††^	633 (442–860)	32.3 (22.5–43.8)
**Race and ethnicity^¶¶^**						
Black or African American	1,381 (1,116–1,704)	4.5 (3.6–5.5)	605 (463–756)	43.8 (33.5–54.7)	NR***	NR***
White	5,811 (5,233–6,450)	3.7 (3.3–4.1)	3,503 (3,216–3,779)	60.3 (55.3–65.0)^††^	1,725 (1,510–1,940)	49.2 (43.1–55.4)
Hispanic or Latino	1,337 (1,051–1,698)	3.0 (2.4–3.8)	611 (464–764)	45.7 (34.7–57.2)	NR***	NR***
Other or multiple races	838 (601–1,164)	3.7 (2.6–5.1)	NR***	NR***	NR***	NR***
**Education**						
Any college	4,764 (4,228–5,366)	2.9 (2.6–3.3)**	2,463 (2,167–2,756)	51.7 (45.5–57.8)	1,061 (887–1,242)	43.1 (36.0–50.4)
No college	4,603 (4,064–5,209)	4.9 (4.3–5.6)**	2,704 (2,424–2,973)	58.8 (52.7–64.6)	1,292 (1,088–1,498)	47.8 (40.2–55.4)
**Employment status**						
Employed full time or part time	3,884 (3,426–4,402)	2.6 (2.3–2.9)**	2,450 (2,191–2,691)	63.1 (56.4–69.3)**	1,287 (1,087–1,483)	52.5 (44.4–60.5)**
Unemployed or other employment**^†††^**	5,483 (4,903–6,126)	5.2 (4.7–5.8)**	2,717 (2,405–3,029)	49.6 (43.9–55.3)**	1,066 (886–1,257)	39.2 (32.6–46.3)**
**Ever arrested and booked**						
Yes	3,620 (3,166–4,130)	9.7 (8.5–11.0)**	2,400 (2,134–2,639)	66.3 (59.0–72.9)**	1,512 (1,316–1,691)	63.0 (54.8–70.5)**
No	5,440 (4,856–6,093)	2.5 (2.3–2.8)**	2,570 (2,273–2,871)	47.2 (41.8–52.8)**	781 (628–953)	30.4 (24.4–37.1)**
**Poverty level^§§§^**						
<100% of the federal poverty level	2,708 (2,335–3,134)	7.5 (6.5–8.7)**	1,530 (1,306–1,744)	56.5 (48.3–64.4)	674 (529–826)	44.1 (34.6–54.0)
100%–199% of the federal poverty level	2,487 (2,125–2,908)	5.0 (4.3–5.8)**	1,540 (1,349–1,718)	61.9 (54.2–69.1)	714 (570–862)	46.4 (37.0–56.0)
≥200% of the federal poverty level	4,172 (3,658–4,756)	2.5 (2.1–2.8)**	2,097 (1,821–2,372)	50.3 (43.6–56.9)	964 (791–1,142)	46.0 (37.7–54.5)
**U.S. Census Bureau region** ^¶¶¶^						
Midwest	2,045 (1,727–2,419)	3.9 (3.3–4.6)	1,084 (892–1,272)	53.0 (43.6–62.2)	478 (370–592)	44.1 (34.2–54.6)
Northeast	1,620 (1,310–2,000)	3.6 (2.9–4.4)	1,020 (851–1,172)	63.0 (52.5–72.3)	523 (404–640)	51.2 (39.6–62.7)
South	3,745 (3,277–4,278)	3.8 (3.3–4.4)	2,083 (1,836–2,322)	55.6 (49.0–62.0)	1,024 (849–1,201)	49.2 (40.8–57.7)
West	1,956 (1,626–2,352)	3.2 (2.7–3.9)	980 (792–1,167)	50.1 (40.5–59.6)	327 (229–442)	33.4 (23.4–45.1)
Core-based statistical area****						
Metropolitan statistical area	7,789 (7,094–8,550)	3.5 (3.2–3.8)**	4,225 (3,848–4,596)	54.2 (49.4–59.0)	1,975 (1,721–2,233)	46.7 (40.7–52.8)
Micropolitan statistical area or outside core-based statistical area	1,578 (1,305–1,904)	4.7 (3.9–5.7)**	942 (789–1,084)	59.7 (50.0–68.7)	377 (278–487)	40.1 (29.5–51.7)
**Overall self-rated health**						
Excellent or very good	2,333 (1,956–2,781)	1.7 (1.4–2.1)**	1,544 (1,336–1,728)	66.2 (57.3–74.1)**^††††^**	790 (623–954)	51.1 (40.3–61.8)
Good	3,600 (3,138–4,125)	4.4 (3.8–5.0)**	2,019 (1,769–2,260)	56.1 (49.1–62.8)	899 (741–1,063)	44.5 (36.7–52.7)
Fair or poor	3,434 (2,984–3,945)	8.8 (7.7–10.2)**	1,604 (1,349–1,864)	46.7 (39.3–54.3)**^††††^**	664 (527–810)	41.4 (32.8–50.5)
**Any mental illness in past year^§§§§^**						
Yes	5,348 (4,844–5,900)	9.0 (8.2–10.0)**	2,957 (2,656–3,251)	55.3 (49.7–60.8)	1,362 (1,154–1,575)	46.1 (39.0–53.3)
No	4,019 (3,525–4,580)	2.0 (1.8–2.3)**	2,210 (1,944–2,469)	55.0 (48.4–61.5)	991 (814–1,173)	44.8 (36.8–53.1)
**Used illicit drugs other than opioids in past year^¶¶¶¶^**						
Yes	4,827 (4,275–5,445)	7.6 (6.7–8.6)**	2,955 (2,630–3,261)	61.2 (54.5–67.6)**	1,562 (1,347–1,774)	52.9 (45.6–60.0)**
No	4,540 (4,026–5,117)	2.4 (2.1–2.7)**	2,212 (1,949–2,476)	48.7 (42.9–54.6)**	790 (636–960)	35.7 (28.8–43.4)**
Binge drinking in past month*****						
Yes	2,366 (1,986–2,815)	3.9 (3.3–4.7)	1,366 (1,148–1,573)	57.7 (48.5–66.5)	585 (442–737)	42.8 (32.4–53.9)
No	7,001 (6,320–7,751)	3.6 (3.2–4.0)	3,801 (3,443–4,152)	54.3 (49.2–59.3)	1,768 (1,539–2,000)	46.5 (40.5–52.6)
**Used marijuana in past year**						
Yes	4,108 (3,596–4,686)	7.0 (6.1–7.9)**	2,534 (2,223–2,824)	61.7 (54.1–68.7)**	1,344 (1,144–1,541)	53.1 (45.1–60.8)**
No	5,259 (4,709–5,871)	2.7 (2.4–3.0)**	2,633 (2,347–2,918)	50.1 (44.6–55.5)**	1,008 (837–1,191)	38.3 (31.8–45.2)**
**Misused central nervous system stimulants in past year^†††††^**						
Yes	2,057 (1,750–2,402)	20.7 (17.6–24.2)**	1,248 (1,042–1,438)	60.7 (50.6–69.9)	741 (603–869)	59.4 (48.3–69.6)**
No	7,310 (6,644–8,041)	3.0 (2.7–3.3)**	3,919 (3,564–4,268)	53.6 (48.8–58.4)	1,611 (1,373–1,861)	41.1 (35.0–47.5)**
**Type of opioid use in past year (among past-year users)^§§§§§^**						
Misused	3,059 (2,735–3,400)	35.9 (32.1–39.9)**	1,562 (1,353–1,771)	51.1 (44.2–57.9)	964 (838–1,080)	61.7 (53.6–69.1)**
Used but did not misuse	5,850 (5,671–7,014)	9.4 (8.4–10.4)**	3,169 (2,831–3,500)	54.2 (48.4–59.8)	1,135 (937–1,348)	35.8 (29.6–42.5)**
**Substance use disorder other than OUD in past year** ^¶¶¶¶¶^						
Yes	4,551 (4,047–5,110)	10.4 (9.3–11.7)**	2,565 (2,280–2,841)	56.4 (50.1–62.4)	1,328 (1,126–1,528)	51.8 (43.9–59.6)**
No	4,816 (4,298–5,394)	2.3 (2.0–2.5)**	2,602 (2,331–2,868)	54.0 (48.4–59.5)	1,025 (851–1,209)	39.4 (32.7–46.5)**
OUD severity******						
Severe	1,384 (1,115–1,717)	100**^††††††^**	734 (587–878)	53.0 (42.4–63.4)**	593 (522–644)	80.7 (71.1–87.7)**
Mild or moderate	4,467 (3,925–5,084)	100**^††††††^**	917 (729–1,140)	20.5 (16.3–25.5)**	509 (405–608)	55.5 (44.1–66.3)**
Did not meet criteria for an active OUD in past year	3,515 (3,082–4,009)	1.4 (1.2–1.6)	3,515 (3,141–3,906)	100**^,^**^§§§§§§^**	1,251 (1,029–1,492)	35.6 (29.3–42.4)**

**Abbreviations:** DSM-IV = *Diagnostic and Statistical Manual of Mental Disorders, Fourth Edition*; DSM-5 = *Diagnostic and Statistical Manual of Mental Disorders, Fifth Edition*; NR = not reported; OUD = opioid use disorder.

* In thousands; estimates are weighted.

^†^ Adults who needed OUD treatment were defined as those who met DSM-5 criteria for OUD or received OUD treatment in the past year.

^§^ Among adults who needed OUD treatment. Adults were classified as having received OUD treatment in the past year if they met any of the following criteria: 1) received inpatient treatment for opioid use, outpatient treatment for opioid use, or medications for OUD in the past year, 2) received inpatient or outpatient treatment in the past year for a substance that they did not specify in the survey, and had past-year opioid use, or 3) did NOT receive inpatient or outpatient substance use treatment, but received substance use treatment virtually or in a prison or jail for an unspecified substance, and had past-year opioid use.

^¶^ Among those who needed and received any OUD treatment. Medication for OUD was defined as having been prescribed medication in the past 12 months to cut back or stop the use of opioids.

** Statistical tests indicate differences between all groups at the 0.05 significance level.

^††^ Statistical tests indicate differences for this group relative to other groups at the 0.05 significance level.

^§§^ Statistical tests indicate differences between those aged 18–25 and 26–34 years at the 0.05 significance level.

^¶¶^ Persons of Hispanic or Latino (Hispanic) origin might be of any race but are categorized as Hispanic; all racial groups are non-Hispanic.

*** Estimate was not reported because of low precision.

^†††^ Other employment includes students, persons keeping house or caring for children full time, retired or disabled persons, or other persons not in the labor force.

^§§§^ Poverty level indicates a person's family income relative to poverty thresholds. The U.S. Census Bureau assigns a poverty threshold for each combination of family size and number of children in the household. The measure excludes persons aged 18–22 years living in a college dorm.

^¶¶¶^ https://www2.census.gov/geo/pdfs/maps-data/maps/reference/us_regdiv.pdf

**** The core-based statistical areas are classifications for all U.S. counties based on the March 2020 core-based statistical area classification provided by the Office of Management and Budget.

^†††† ^Statistical tests indicate differences between excellent or very good and fair or poor at the 0.05 significance level.

^§§§§^ Any mental illness aligns with DSM-IV criteria and is defined as having a diagnosable mental, behavioral, or emotional disorder, other than a developmental or substance use disorder. These mental illness estimates are based on a predictive model and are not direct measures of diagnostic status.

^¶¶¶¶^ Illicit drug use other than opioid use includes the use of cocaine, hallucinogens, inhalants, marijuana, or methamphetamine, or misuse of prescription tranquilizers, sedatives, or stimulants.

***** Defined for females as drinking four or more drinks on the same occasion and for males as drinking five or more drinks on the same occasion on ≥1 day in the past 30 days.

^†††††^ Central nervous system stimulant misuse includes use the use of cocaine or methamphetamine or misuse of prescription stimulants.

^§§§§§ ^Misuse includes use of heroin or use of prescription pain relievers in any way not directed by a health care professional. “Use but not misuse” was defined as using only prescription pain relievers as directed by a health care professional.

^¶¶¶¶¶^ Includes use disorders for alcohol, cocaine, hallucinogens, inhalants, marijuana, methamphetamine, prescription sedatives, prescription stimulants, or prescription tranquilizers.

****** OUD severity level is determined by the number of individual DSM-5 criteria met for OUD. Mild or moderate OUD means two to five criteria were met. Severe OUD means six or more criteria were met. Persons who did not meet criteria for an active OUD in the past year and received OUD treatment in the past year were assumed to have had an active OUD in the past and to have successfully treated OUD with continued treatment.

^††††††^ By definition, 100% of adults with OUD or who received OUD treatment were classified as needing OUD treatment.

^§§§§§§^ By definition, among adults who 1) did not meet the criteria for an active OUD based on past-year symptoms and 2) were classified as needing OUD treatment in the past year; 100% received OUD treatment.

### Need for OUD Treatment

The percentage of adults aged 18–25 years who needed OUD treatment (2.2%) was lower than that among older age groups (range = 3.7%–4.3%) (Table). Groups in which a high percentage of persons needed OUD treatment included those who did not attend college (4.9%), were not employed (5.2%), or had ever been arrested and booked (9.7%). Need for OUD treatment increased with poverty level: it was lowest (2.5%) among those with income ≥200% of the federal poverty level [FPL], increasing to 5.0% among those with income 100%–199% of FPL, and was highest (7.5%) among persons with income <100% of FPL. The percentage of adults who needed OUD treatment was elevated among those who, during the previous year, had any mental illness (9.0%), used illicit drugs other than opioids** (7.6%) or marijuana (7.0%), misused stimulants^††^ (20.7%) or opioids^§§^ (35.9%), or had a nonopioid SUD (10.4%).

### OUD Treatment

The percentage of adults needing OUD treatment who received treatment was lower among those aged ≥50 years (44.9%) than among younger age groups (range = 58.9%–67.8%) (Table). The percentage of adults who received treatment was higher among non-Hispanic White (White) adults (60.3%) than among non-Hispanic Black or African American (Black) (43.8%) or Hispanic or Latino (Hispanic) (45.7%) adults and among adults with severe OUD (53.0%) than among those with mild or moderate OUD (20.5%). Compared with other adults, the percentage who received OUD treatment was higher among those who were employed (63.1%), had ever been arrested and booked (66.3%), or had used illicit drugs other than opioids (61.2%).

### Receipt of Medications for OUD

Among adults who needed and received any OUD treatment, fewer than one half (45.5%) received medications for OUD. The percentage of adults who received medications for OUD was higher among those who were employed (52.5%), were ever arrested and booked (63.0%), used illicit drugs other than opioids (52.9%), used marijuana (53.1%), misused stimulants (59.4%), misused opioids (61.7%), or had a past-year SUD involving a substance other than opioids (51.8%), than among those without these characteristics or exposures. The percentage who received medications for OUD was higher for men (51.0%) than for women (39.5%), for those aged 35–49 years (68.4%) than for those in other age groups (18–25 = 19.9%; 26–34 = 44.1%; ≥50 = 32.3%), and for those with severe OUD (80.7%) than for those with mild or moderate OUD (55.5%).

## Discussion

Among adults needing OUD treatment in 2022, only 25% received medications for OUD; 30% received OUD treatment not including these medications. These findings underscore disparities in treatment and a need to increase use of medications for OUD. Lower percentages of Black and Hispanic adults, who have been particularly affected by increasing overdose deaths (*3*), received any OUD treatment compared with White adults. Among adults who received OUD treatment, lower percentages of women and younger and older adults received medication. Higher proportions of persons with other drug use or misuse or who had ever been arrested and booked received medications for OUD; these findings might reflect greater awareness of treatment need or contact with systems linking persons to OUD treatment. Higher percentages receiving medication among adults with severe OUD might reflect perception or more clinician recognition of treatment need among adults with six or more OUD symptoms. Still, among adults with severe OUD, fewer than one half (80.7% of the 53.0% who received any OUD treatment) received medications for OUD, underscoring the large gap in receipt of evidence-based treatment, even for this highly affected group.

Approximately 43% of adults needing OUD treatment did not perceive that they needed it, consistent with previous findings that large proportions of persons with SUDs did not feel that they needed treatment.^¶¶^ Patients taking opioids only as prescribed (who constitute a majority of persons meeting OUD criteria***) might be particularly unlikely to perceive a need for OUD treatment, even if they experience OUD symptoms. If clinicians suspect that patients prescribed opioids for pain have OUD on the basis of patient concerns or behaviors, or if patients experience harm from opioids or choose to but are unable to taper opioids, clinicians should discuss their concern with the patient, provide an opportunity for the patient to disclose related concerns or problems, and assess for OUD using DSM-5 criteria (*4*). Nonjudgmental support and harm reduction approaches can establish rapport, build trust, and reduce overdoses and other harms among persons not ready for treatment.^†††^

Several factors limit access to medications for OUD despite strong recommendations for their use (*4**,**5*). Some clinicians prefer an approach that does not include medications, and some hold beliefs equating medications for OUD with illegal substance use (*6*). Methadone for OUD can only be dispensed from a Substance Abuse and Mental Health Services Administration–certified opioid treatment program (OTP); many U.S. counties have no OTP.^§§§^ Buprenorphine or naltrexone can be prescribed in any setting, but several barriers exist. Many facilities treating OUD do not offer these medications; some do not accept clients using medications for OUD.^¶¶¶^ In addition, large proportions of pharmacies do not stock buprenorphine.**** Payors, including many state Medicaid programs, have restrictions (such as prior authorization) that can delay dispensing of some buprenorphine formulations (*7*). Fewer than 10% of physicians^††††^ obtained the waiver that, until 2023, was required to prescribe buprenorphine for OUD. Primary care physicians have reported barriers to obtaining the waiver and prescribing buprenorphine, including too little experience treating OUD, concern about being inundated with requests for buprenorphine, lack of access to addiction or behavioral health specialists, and acquiring the training required to obtain a waiver (*8*).

### Limitations

The findings in this report are subject to at least five limitations. First, the number of persons needing OUD treatment presented in this report are likely underestimates; NSDUH is a household survey, includes persons experiencing homelessness only if they use shelters, and does not include residents of institutional group quarters such as jails. Second, NSDUH response rates in 2021 and 2022 were lower than in previous years, which might increase the potential for nonresponse bias resulting in over- or underestimates. Third, sample size limited some comparisons of OUD treatment across racial and ethnic groups, prohibited comparisons across health insurance coverage, and precluded treatment estimates specific to persons with mild OUD or with moderate OUD. Medications for OUD are strongly recommended, particularly for moderate or severe OUD (*4**,**5*). However, Food and Drug Administration approvals for medications for OUD were based on data for patients with opioid dependence as defined by *Diagnostic and Statistical Manual of Mental Disorders, Fourth Edition*; application to DSM-5–defined mild OUD is less clear (*5*). Understanding specific treatment needs for patients with mild OUD merits further study. Fourth, cross-sectional survey responses were insufficient to ascertain the presence of OUD symptoms before the preceding year. Finally, OUD was a proxy diagnosis based on respondents’ answers to questions corresponding to diagnostic criteria; respondents were not asked whether they had ever received a clinical diagnosis of OUD.

### Implications for Public Health Practice

The shift from use of heroin to illegally manufactured fentanyl has increased the likelihood that overdoses are fatal (*9*), adding urgency to the need to provide effective care for persons with OUD (*2*). This need is particularly acute for Black and Hispanic adults (*3*,*10*), women, and younger and older adults. Population-level interventions across a variety of settings are needed to link persons to care,^§§§§^^,^^¶¶¶¶^ initiate medications for OUD,***** and support sustained treatment and recovery.

Expanded communication about effectiveness of medications for OUD is needed to reduce nonfatal and fatal overdoses. Increasing awareness among persons who use drugs and their families, friends, and other contacts that medications for OUD are effective is critical.^†††††^ Clinicians and treatment providers should offer or arrange evidence-based treatment, including medications for OUD (*4*). As of 2023, a waiver is no longer required to prescribe buprenorphine. All clinicians with a current Drug Enforcement Administration registration including Schedule III authority may prescribe buprenorphine for OUD if permitted by applicable state law.^§§§§§^ Guidance (*4*,*5*) and mentoring^¶¶¶¶¶^ are available for diagnosis and management of opioid use disorder. Pharmacists and payors can work to make these life-saving medications available without delays.

## Footnotes

*
https://www.cdc.gov/nchs/data/databriefs/db491-tables.pdf

†
https://www.samhsa.gov/data/report/nsduh-2022-methodological-resource-book-mrb

§
https://dsm.psychiatryonline.org/doi/book/10.1176/appi.books.9780890425596

¶ 45 C.F.R. part 46.102(l)(2), 21 C.F.R. part 56; 42 U.S.C. Sect. 241(d); 5 U.S.C. Sect. 552a; 44 U.S.C. Sect. 3501 et seq.

** Illicit drug use other than opioid use includes the use of cocaine, hallucinogens, inhalants, marijuana, or methamphetamine, or misuse of prescription tranquilizers, sedatives, or stimulants.

†† Stimulant misuse includes the use of cocaine or methamphetamine or use of prescription stimulants in any way not directed by a health care professional.

§§ Opioid misuse includes use of heroin or use of prescription pain relievers in any way not directed by a health care professional.

¶¶
https://www.samhsa.gov/data/sites/default/files/reports/rpt29393/2019NSDUHFFRPDFWHTML/2019NSDUHFFR090120.htm

***
https://www.samhsa.gov/data/report/2021-nsduh-annual-national-report

†††
https://store.samhsa.gov/product/tip-63-medications-opioid-use-disorder/pep21-02-01-002

§§§
https://www.everycrsreport.com/files/20190624_R45782_ed39091fadf888655ebd69729c3180c3f7e550f6.pdf

¶¶¶
https://www.samhsa.gov/data/sites/default/files/reports/rpt35313/2020_NSSATS_FINAL.pdf

****
https://www.samhsa.gov/sites/default/files/policy-priority-roundtable-buprenorphine-access-pharmacies.pdf

††††
https://aspe.hhs.gov/sites/default/files/documents/facbce1704035fded1034192d148304d/buprenorphine-practice-guideline-early-impacts.pdf

§§§§
https://stacks.cdc.gov/view/cdc/119464

¶¶¶¶
https://www.cdc.gov/overdose-prevention/php/od2a/prevention.html

*****
https://www.samhsa.gov/sites/default/files/grants/pdf/fy-2024-sor-nofo.pdf

†††††
https://pcssnow.org/courses/guide-for-families-medications-for-opioid-use-disorder-2/

§§§§§
www.samhsa.gov/medications-substance-use-disorders/waiver-elimination-mat-act

¶¶¶¶¶ https://pcssnow.org/mentoring/
